# Response to “Letter to the editor RE: “Conservative management of 11 weeks old cervical ectopic pregnancy with transvaginal ultrasound-guided combined methotrexate injection: Case report and literature review””

**DOI:** 10.1016/j.ijscr.2020.09.015

**Published:** 2020-09-23

**Authors:** Ipek Betul Ozcivit, Ismail Cepni, Kubra Hamzaoglu, Hakan Erenel, Rıza Madazlı

**Affiliations:** Department of Obstetrics and Gynecology, Cerrahpasa Medical Faculty, Istanbul University Cerrahpasa, Istanbul, Turkey

Dear Editor,

We appreciate the comments of Dr. Naem and Dr. Al-Kurdy. This case report emphasizes that local methotrexate injection may become the treatment of choice for cervical ectopic pregnancies in the near future. Injection of methotrexate in a cervical pregnancy with advanced gestational age (11 w) and CRL of 41 mm makes this case unique. This is the most advanced cervical pregnancy in the literature where methotrexate has been administered and resolved the pregnancy without further intervention [[2], [3], [4], [5]]. The complications observed in the previous cases were sepsis and need of blood transfusion, the second dose of methotrexate, cervical tamponade and embolization to stop the vaginal bleeding, secondary surgery and interval cervical curettage.

As Dr. Naem and Al-Kurdy mentioned, it is usually the main concern of the patient to preserve her fertility [1]. Conservative methods like methotrexate administration provide the patient the chance to preserve her fertility. Similar cases in the literature reported patients treated with methotrexate have favorable obstetric outcomes in their subsequent pregnancies, regardless the required additional treatment [6]. Further studies are necessary to compare the obstetric outcomes between patients treated with methotrexate alone and methotrexate plus required additional treatment.

Our patient had almost a symptom free follow up after methotrexate injection, so on the 7th day of her procedure she was offered to discharge with the intention of decreasing hospital stay. The pattern of the hCG decline in our patient, where hCG values normalized after 90 days from the injection, was similar with the other studies [4,5].

Success of systematic methotrexate injection is related to absence of fetal cardiac activity, level of hCG (<10,000 mIU/mL), gestational age smaller than 9 weeks and a crown-rump length(CRL) of <10 mm [7]. Yet, we preferred both intraamniotic and systemic management due to the high levels of hCG in our case. We believe that the fixed pressure in the gestational sac, which was achieved by giving the same amount of fluid as aspirated, provided a complication free recovery. Due to less quantity of muscular tissue in cervix, preserving constant pressure may be the key factor to prevent major amount of bleeding.

Overall, local methotrexate injection is a safe method to practice in cervical pregnancies with minimum complication risk, regardless the gestational age, presence of fetal cardiac activity and CRL.

## Declaration of Competing Interest

None.

## Funding

None.

## Ethical approval

None. Our paper is in the format of letter to editor.

## Consent

None. Our paper is in the format of letter to editor.

## Author contribution

Our paper is in the format of letter to editor. It is a response to the letter to editor which referred to our article “Conservative management of 11 weeks old cervical ectopic pregnancy with transvaginal ultrasound-guided combined methotrexate injection: Case Report and Literature Review” written by the authors listed below:

Ipek Betul Ozcivit, MD.

Ismail Cepni, Prof. MD.

Kubra Hamzaoglu, MD.

Hakan Erenel, MD.

Rıza Madazlı, Prof. MD.

## Registration of research studies

N/A.

## Guarantor

Ismail Cepni, Prof. MD.
